# Label-free biosensing of *Salmonella enterica* serovars at single-cell level

**DOI:** 10.1186/s12951-017-0273-6

**Published:** 2017-05-17

**Authors:** Bin Wang, Bosoon Park, Bingqian Xu, Yongkuk Kwon

**Affiliations:** 1 Single Molecule Study Laboratory, College of Engineering and Nanoscale Science and Engineering Center, University of Georgia, Athens, GA 30602 USA; 2 USDA-ARS, U.S. National Poultry Research Center, Athens, GA 30605 USA; 3 Animal and Plant Quarantine Agency, Gimcheon, Republic of Korea

**Keywords:** Atomic force spectroscopy, Single-molecule, Force microscopy, Surface plasmon resonance, *Salmonella*, Bacteria

## Abstract

**Background:**

The emerging nanotechnologies have greatly facilitated the development of label-free biosensors. The atomic force microscopy (AFM) has been used to study the molecular mechanism of the reactions for protein and aptamers. The surface plasmon resonance (SPR) have been used in fast detections of various pathogens such as bacteria. This study used both AFM and SPR to investigate the complex reactions between aptamers and outer membrane proteins (OMPs) on the surface of *S. typhimurium*.

**Results:**

Two DNA aptamers were used for the label-free detections of *S. typhimurium* by AFM and SPR. The aptamers have specific binding affinities to the OMPs of *S. typhimurium*. At single-molecule level, the high resolution AFM topography and recognition images distinguished the OMPs on the bacteria surface, which is the first time the location of individual outer membrane protein have been determined on *Salmonella* surface. *E. coli* in the control experiments didn’t generate recognition signals, which proved the specificity of these two aptamers to *S. typhimurium*. The off-rate values for the interactions of these two aptamers to the OMPs were estimated as 5.2 × 10^−3^ and 7.4 × 10^−3^ s^−1^, respectively, by the AFM dynamic force microscopy (DFS). The force and extension values form DFS measurements were used to distinguish the two aptamers. The surface membrane model was proposed to explain the complex correlations among force and extension values. Next, these two aptamers were used in the bulk solution detections of *S. typhimurium*. The gold chips in SPR experiments were modified with carboxymethylated-dextran (CD), followed by aptamers immobilization, to reduce the non-specific binding signals. The limit of detection (LOD) was determined as 3 × 10^4^ CFU mL^−1^.

**Conclusions:**

The AFM single-molecule study revealed detailed information about the unbinding force and extension of the aptamer in complex biological reactions. The careful analysis of the experimental results provide better understanding of the molecular mechanism of OMPs reactions. The single-molecule measurements are helpful in evaluating the specificity of binding reagents, such as aptamers, in bulk solution detections. The protocols used in the SPR detections can be expanded into the label-free detections of other bacterial pathogens.

## Background

The food industry is consistently facing the challenge for detection of various toxic species that can contaminate food products [1]. Among different foodborne pathogens, *Salmonella enterica* is one of the major causes of gastrointestinal infections in human and animals, and cause hundreds of death every year [2, 3]. The detection of *Salmonella enterica* serovars from food sources is critical for the prevention and control of the outbreak of salmonellosis. With the emerging nanotechnologies, new label-free detection methods have been developed for the fast detections of biological species with high sensitivity and specificity [4].

In microbial studies, both label-based and label-free techniques have been used for bio-imaging or bio-sensing purposes. For the label-free techniques, the detection signals are generated from endogenous materials and properties. They are particularly useful in the direct monitoring of living cells where label-based techniques have shown drawbacks that sometimes can become critical for the accurate detections. The labelling processes may induce unwanted side effects in the biological samples, especially in the living cells, and in turn affect the sensitivity and selectivity in quantitative studies. Moreover, the labelling methods require additional time, labor, and skills to obtain the specific sample signals while maintaining the activity of the biomolecules or cells. With the rapid development of nanotechnology, label-free detections have reached down to the nanometer scale, opening new opportunities in the interdisciplinary fields of single-cell or single-molecule microbiology. The atomic force microscopy (AFM) is a versatile platform to be used for both surface imaging and force sensing. The ultra-high resolution and sensitivity make AFM a powerful tool for label-free detections and overcome those challenges of label based detection methods.

The label-free biosensors based on nanotechnologies include bulk solution methods such as surface plasmon resonance (SPR), and single-molecule methods such AFM [5]. The SPR technology has the advantages of easily operation and fast detection [6, 7]. Although SPR instruments have been used in the detections of bacteria samples, the dynamic range and the limit of detection (LOD) of current SPR methods still need a lot of improvements [8–10]. Especially, different serotypes of *Salmonella* have shown the preference to their specific host species [2]. The detection of those host-specific *Salmonella* serotypes has become an important issue for the food industry and disease control. Most methods used for *Salmonella* serotyping are based on genetic analysis, but the fast detections with antibodies or aptamers have drawn increasing attention recently [11, 12].

Here the anti-*Salmonella* aptamers were used as probe molecules to specifically detect *S. typhimurium*. The aptamers #33 (APT33) and #45 (APT45) have specific interaction to the *Salmonella* outer membrane proteins [3]. Aptamers have shown promising properties as the probe molecules for the detections of various biospecies [13]. It is relatively easy to attach functional linker and spacer molecules to aptamer sequences, and immobilize the aptamers to the biosensor substrate [14, 15]. However, it is important to test the activity of aptamers to their target species after the immobilization step [5]. In this work, the gold substrate of the biosensor was chemically modified to immobilize the aptamer for both SPR and AFM measurements [5]. The *S. typhimurium* water solution was used as sample solution, and the interactions between the bacteria and the aptamers on the substrate were carefully investigated by both single-molecule (AFM) and bulk solution (SPR) measurements.

Among various nanotechnologies, the AFM techniques provide the highest resolution among all surface detection methods. The AFM topography and recognition images of the biosensor showed the morphology of *S. typhimurium*, and the AFM recognition images can provide information about the locations of the single-molecule interactions of the bacteria to the aptamers attached on the AFM tip [13]. The AFM dynamic force spectroscopy (DFS) can be used to quantitatively measure the detailed and complex single-molecule interaction forces between the probe molecule aptamer and the target species, such as *S. typhimurium*. Therefore, this single-molecule method can be used to test the affinity of the aptamers integrated in the biosensor device, providing the critical guide for the design of bulk solution sensors, such as SPR [5]. On the other hand, the SPR measurements showed the quantitative detection of *S. typhimurium* under different concentrations. The comparison of the quantitative measurements from these two platforms is important for the development of biosensors. When the dimensions of biosensors go down to nanometer scale, the biosensor components, such as the surface modification, linker molecules, and probe molecules, will show their unique physical and chemical properties, which may be different from the ones in large scale measurements [16–18]. Therefore, AFM is a versatile platform to detect and distinguish these unique biosensor properties in the nanometer scale. The combination of SPR and AFM will provide critical reference for the future development of biosensors in nanometer scale.

## Methods

### Materials

The aptamer sequences against *S. typhimurium* were obtained from literature [3]. Two sequences with high affinities to *S. typhimurium* were APT33, 5′-TATGG-CGGCG-TCACC-CGACG-GGGAC-TTGAC-ATTAT-GACAG-3′, and APT45, 5′-GAGGA-AAGTC-TATAG-CAGAG-GAGAT-GTGTG-AACCG-AGTAA-3′ were selected from random ssDNA sequence pools. In both AFM and SPR experiments, the aptamer sequence was modified by twenty thymine bases as the spacer and the amine group as the linker at 5′ end. The modified sequence was purchased from Integrated DNA Technologies (Coralville, IA, USA). In AFM experiment, the aptamer was attached by its amine group to the linker molecule and in turn to the gold coated tip. The polymer linker thiol-(polyethylene glycol)-acid (HS-PEG-COOH, M.W. 2000) was purchased from Creative PEGWorks (Winston Salem, NC, USA). The cystamine, carboxymethylated-dextran (CD), N-hydroxysuccinimide (NHS), and ethanolamine were all purchased from Sigma-Aldrich (St. Louis, MO, USA). The 1-(3-dimethylaminopropyl)-3-ethylcarbodimide hydrochloride (EDC) was purchased from Flucka Chemicals (Sigma-Aldrich, St. Louis, MO, USA). Phosphate buffer saline (PBS, pH 7.2) was purchased from Pierce (Thermo Scientific, Waltham, MA, USA). Triplet deionized water was provided by a Barnstead Nanopure Diamond Laboratory Water System (Barnstead Thermolyne, Dubuque, IA, USA).

### Apparatus

In the SPR experiment, the sensor film was coated with cystamine self-assembled monolayers (SAMs) via S-Au linkage by spreading a droplet (20 μL) consisting of cystamine hydrochloride (20 mM) onto the Au film overnight at room temperature. Then, the mixture solution of 15 mM EDC, 75 mM NHS, and 10 mg mL^−1^ CD was dropped onto the cystamine modified Au surface for 2.5 h and the Au surface was cleaned with deionized water. After the sensor chip was mounted on the SPR prism with the refractive index matching liquid, 100 μL of the mixture of 15 mM NHS and 75 mM EDC in deionized water was injected into the cuvette for 10 min to activate the carboxyl group of CD. Afterward, the aptamer solution (1 μM in PBS) was injected into the flow cell for its immobilization on the CD modified Au surface. Finally, to deactivate the remaining active sites on the sensor chip surface, 100 μL of 1 M EA in deionized water was injected. With the continuous flushing of the flow cell with deionized water, the *S. typhimurium* solutions at concentrations 7.6 × 10^2^, 7.6 × 10^4^, and 6.2 × 10^6^ CFU mL^−1^ were injected into the BI-2000 SPR system (Biosensing Instrument, Tempe, AZ) for the SPR measurements. Flow rate was controlled at 20 μL per minute and all the sample solutions were injected at the rate of 20 μL per minute with a unified volume of 100 μL.

For AFM experiments, the amine modified aptamer was attached to the thin film of CD on Au surface. The TopMAC mode and PicoTREC module (Agilent Technologies, Santa Clara, CA, USA) were used to obtain the topography images and the recognition images, respectively, in water solutions of *S. typhimurium*. The AFM topography images of *S. typhimurium* in air were also obtained for comparison. However, the aptamer shows specific activity against the bacteria in water during the aptamer selection process. In air and dry condition, the aptamer may change its structure and affinity. Therefore, the structure and activity of this aptamer should be studied in solution, not in air. The aptamer was attached to the gold coated AFM tip with HS-PEG-COOH linker molecule and EDC/NHS coupling reaction. The aptamer molecules were also immobilized on Au(111) surface by the same method as in SPR measurement in order to capture the *S. typhimurium* in the water.

## Results and discussion

### Single-molecule measurements using AFM

**Fig. 1:**
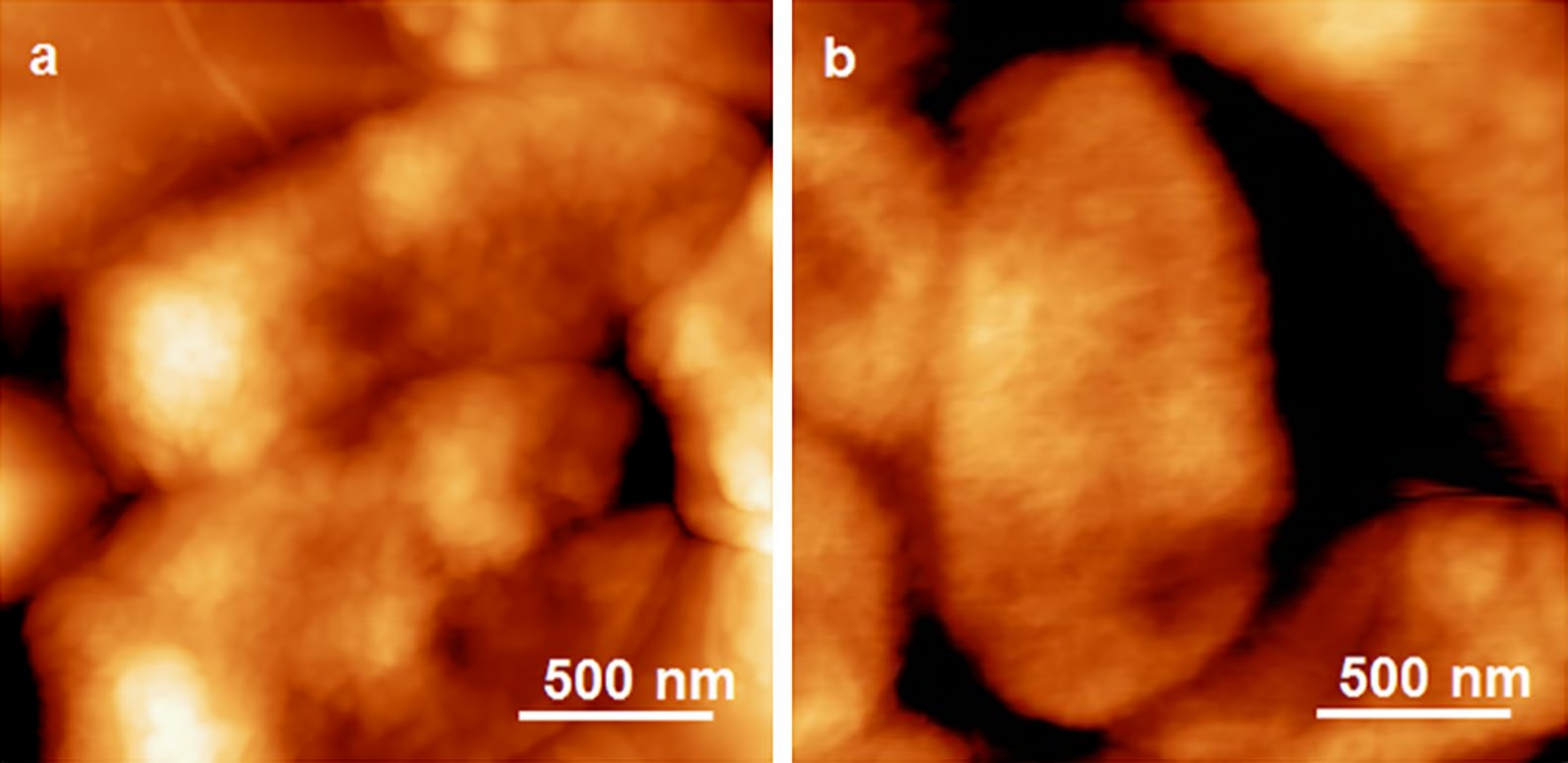
The representative AFM topography image of *S. typhimurium* (**a**) and *E. coli* (**b**), with the image size of 2 μm by 2 μm

The AFM topography image of *S. typhimurium* under dry condition on gold surface is shown in Fig. 1a. This image has higher resolution, because of much less thermal noise in air than in solution, where the aqueous environment has greater interference to the AFM tip and the sample surface. The length of the bacteria is around 2.5 μm, the width is around 1 μm, and the height of bacteria is around 200 nm. The size and shape of *S. typhimurium* in the AFM image are consistent with the ones in other literature, but the topography image itself cannot show the feature of any *Salmonella* serotype [19].

**Fig. 2:**
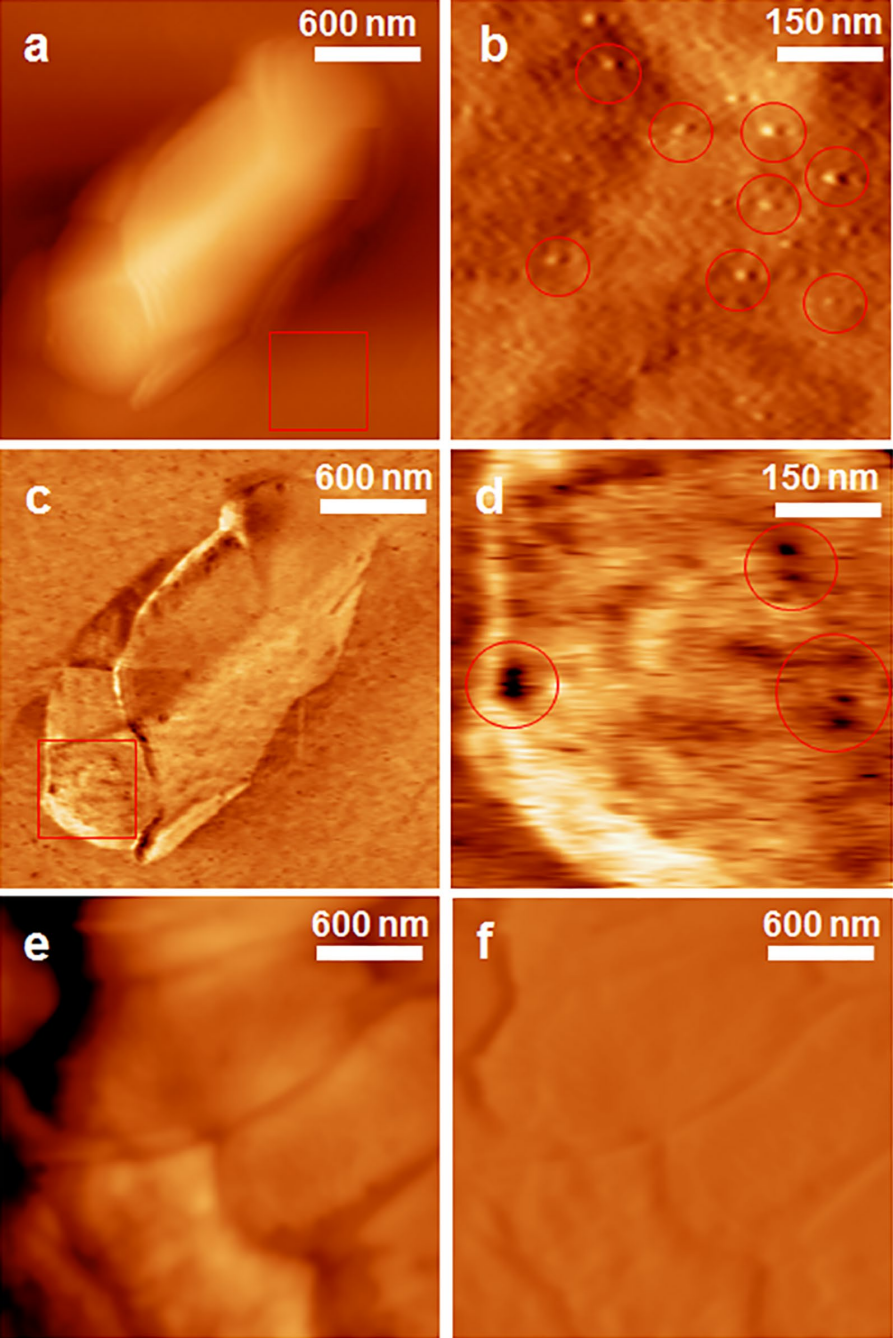
The representative AFM topography and recognition images of *S. typhimurium* and *E. coli* in water, obtained by the APT33 attached to AFM tip and on CD-modified Au(111) surface. **a** The topography images of *S. typhimurium*. **b** The enlarged topography image shows the aptamers on the CD-modified Au(111) surface within the red frame in **a**. Some representative aptamer molecules were highlighted with *red circles*. **c** The corresponding recognition image of *S. typhimurium*. **d** The enlarged recognition image of the surface area within the red frame in **c**. The *dark spots* in the *red circles* show the location of OMPs. **e** The topography image of *E. coli* in water. **f** The corresponding recognition images of *E. coli*. The images of **a**, **c**, **e**, and **f** have the same size of 2.4 μm by 2.4 μm. The images of **b** and **d** have the same size of 600 nm by 600 nm

The AFM topography images of *S. typhimurium* on CD modified Au(111) surface showed the outlines of the entire bacteria body in water (Fig. 2a). The CD modification is a well-developed method to significantly reduce the non-specific interactions between the gold substrate and biological sample [20]. The length of the bacteria is around 2 μm, and the width is around 1 μm. The height of bacteria is around 200 nm, which indicates that the individual bacteria cell was stretched on the gold substrate in x and y directions of the AFM scanning. The area inside the red frame of Fig. 2a was enlarged in Fig. 2b, which shows the aptamer molecules (bright spots) immobilized on CD modified Au(111) surface. The recognition image (Fig. 2c) showed clear recognition signals (black spots) on the bacteria surface. The locations of these recognition signals are corresponding to the locations of the surface proteins that have specific interactions with the aptamer (Fig. 2d). On the other hand, the *E. coli* showed similar morphology as the *S. typhimurium* in water (Fig. 2e), but no clear recognition signals (Fig. 2f). To our best knowledge, this was the first time the locations of individual outer membrane proteins of *S. typhimurium* had been visualized using AFM recognition images. It is intriguing that the recognition signals showed in certain round-shaped small areas with the diameters varying from 10 to 60 nm. One of the possible explanations is that those OMPs concentrated in small outer membrane vesicles (OMVs) on the bacteria surface [21, 22]. The *S. typhimurium* cells can use these OMVs for intercellular communication, transferring materials into host cells, and many other physiological and pathological functions [23, 24]. The OMVs are also involved in protein secretion pathways of gram-negative bacteria, so the OMP molecules inside those OMVs were detected by the aptamer during the AFM scanning and showed strong recognition signals [25]. Therefore, although the topography images show higher resolutions in dry conditions, the topography and recognition images in solution give more useful information for the activity of the bacteria and its OMPs.

The size of the bacteria cell in Fig. 2 is different from the one in Fig. 1. The main reason is that the bacteria in solution might be attached on gold surface along a certain orientation in the liquid. However, the bacteria in air had to lay flat on the gold surface because no liquid existed to support other orientations of the cell body. Therefore, the AFM images in water can reveal more details about the real-time status of the bacteria and its interactions with the aptamer.

**Fig. 3:**
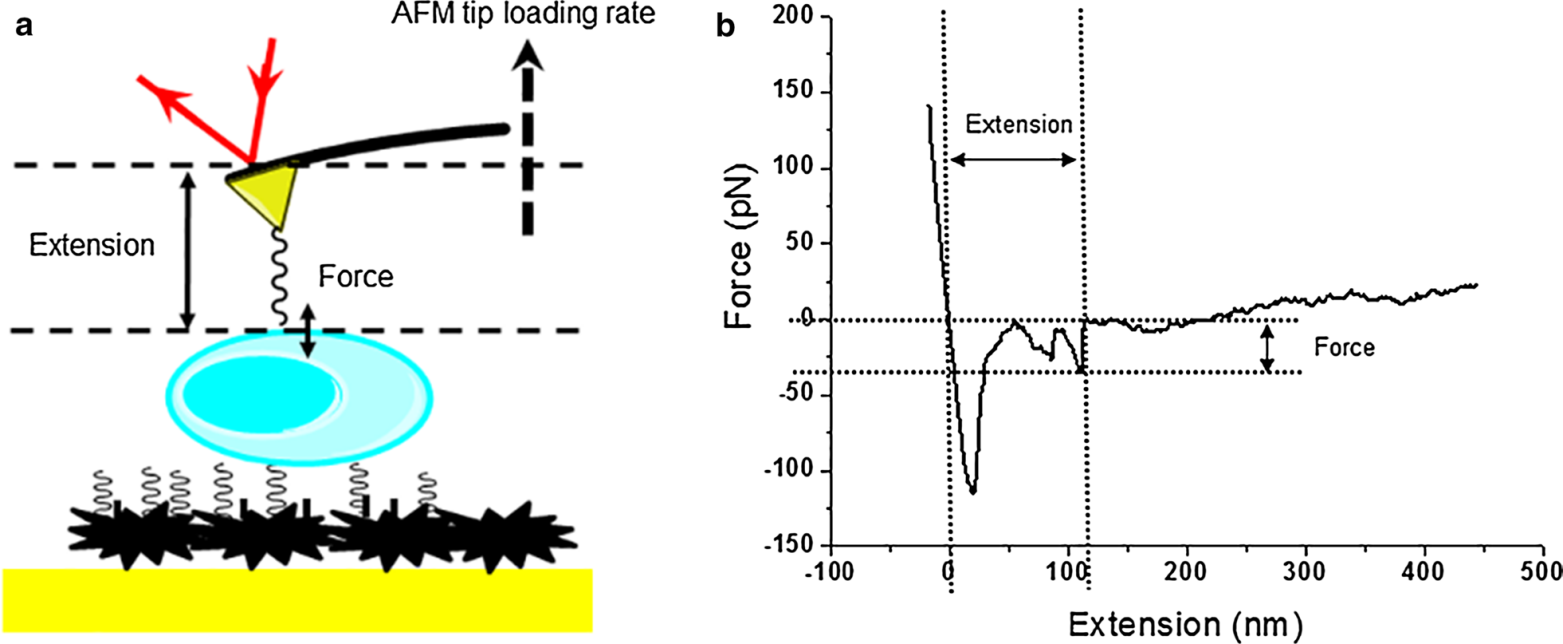
The schematics of DFS measurements on bacteria surface (**a**) and a representative force-extension curve (**b**)

The DFS technique has been proved to be a powerful tool for the study of single-molecule structure–function relationship in previous study [5, 26]. Here the similar methods have been used to investigate the in situ interactions between aptamers and OMPs. The experimental setup is shown in Fig. 3a. The aptamer modified tip was moved into the OMP concentrated area, and the tip will move in the direction perpendicular to the bacteria surface and obtain the force-extension curve. Most curves obtained in the measurements have shown more than one unbinding peak, as shown in Fig. 3b.

**Fig. 4:**
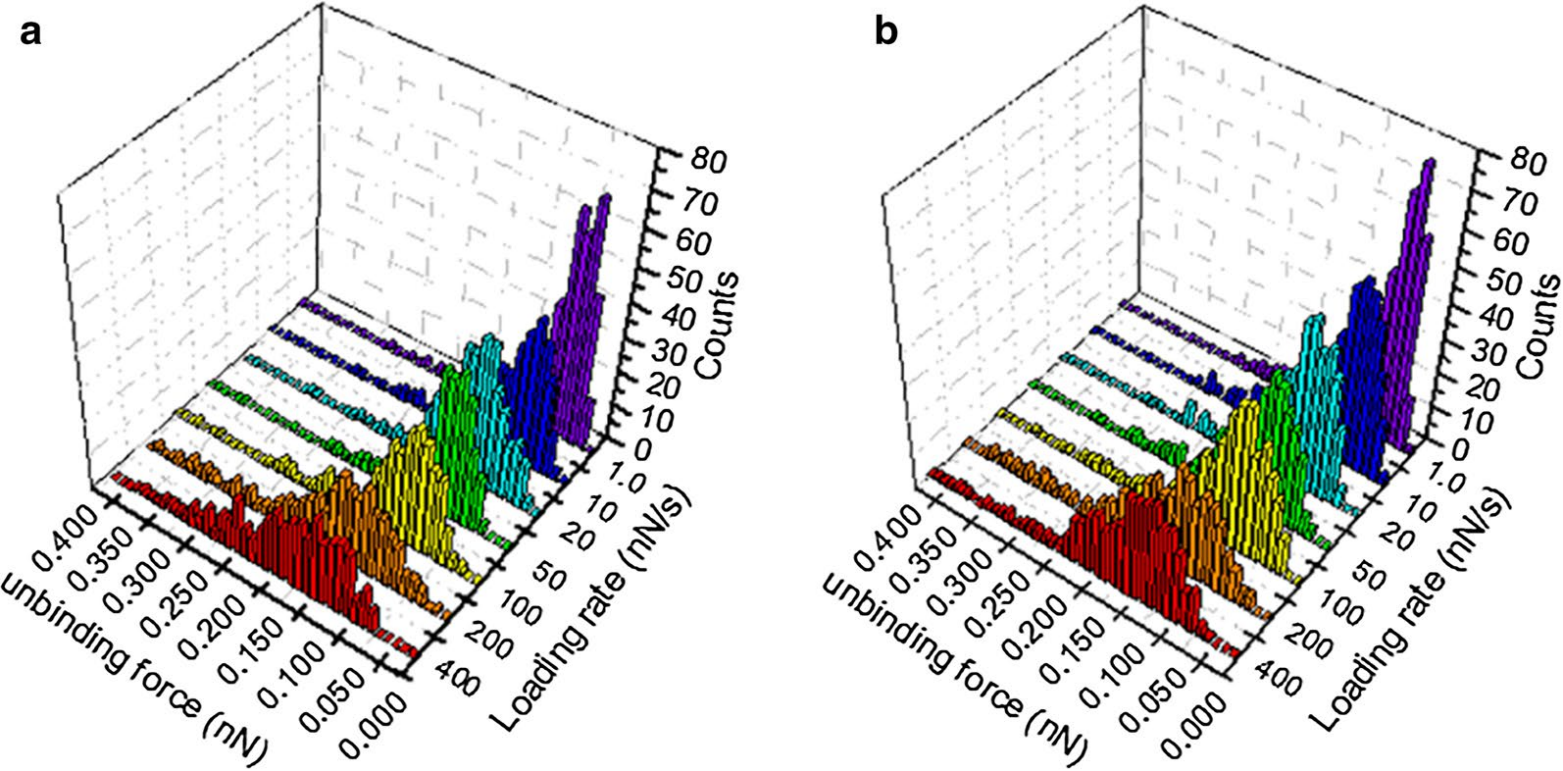
Force histograms for aptamer 33 (**a**) and aptamer 45 (**b**) in the DFS measurements under seven different loading rates

**Fig. 5:**
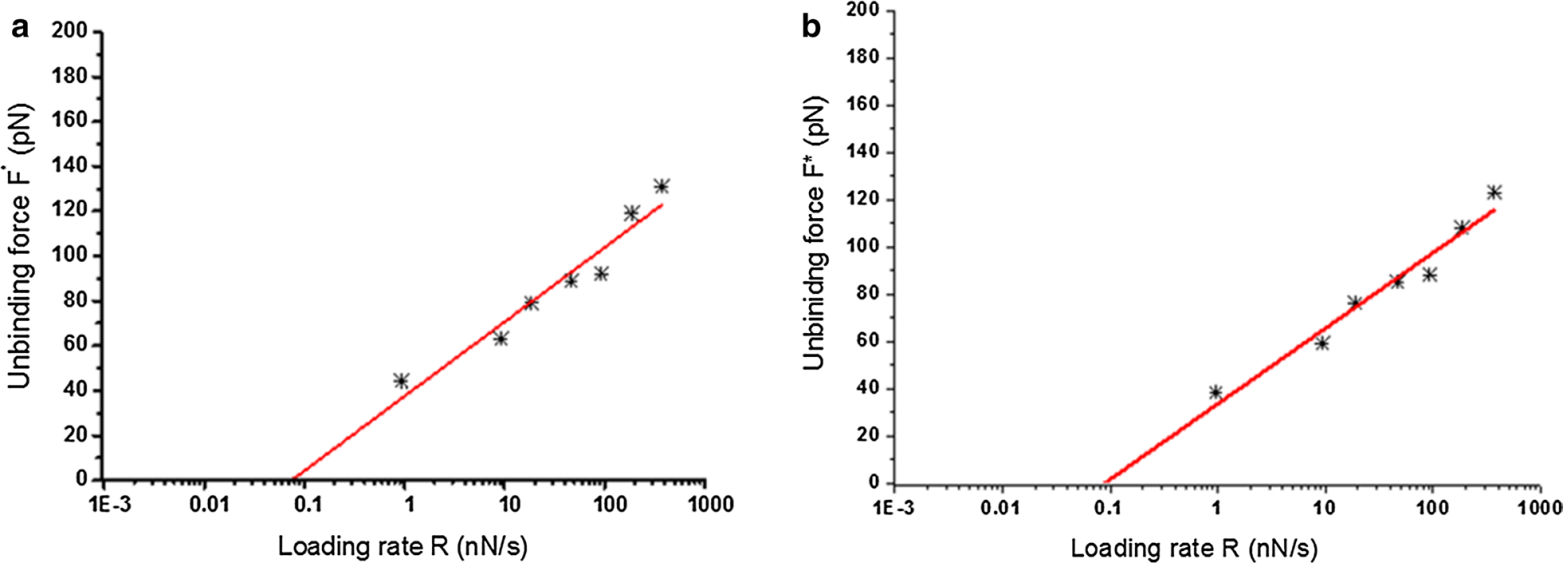
F* vs. LnR plots for aptamer 33 (**a**) and aptamer 45 (**b**) in the DFS measurements under seven different loading rates

The force histograms and extension histograms have been constructed under different loading rates, ranging from 1 to 400 nN s^−1^. Under each loading rate, 300 force-extension curves were collected to construct the force histogram and the extension histogram. Figure 4 shows the force histograms of aptamer 33 (Fig. 4a) and 45 (Fig. 4b) under these seven loading rates (R): 1, 10, 20, 50, 100, 200, and 400 nN s^−1^. Comparing the two aptamers, the force histograms under the same loading rate did not demonstrate any significant differences for the peak that represents the most probable unbinding force (F*). According to Bell’s model, the affinity of APT33 to OMPs (k_off_33) was estimated from the F* vs. LnR plot, and the (k_off_45) was obtained by the same method (Fig. 5). The two off-rate values are very close each other, with 5.2 × 10^−3^ s^−1^ for k_off_33 and 7.4 × 10^−3^ s^−1^ for k_off_45. The results indicate that the affinity measurement may not be able to distinguish these two aptamers for their interactions with *S. typhimurium*.

**Fig. 6:**
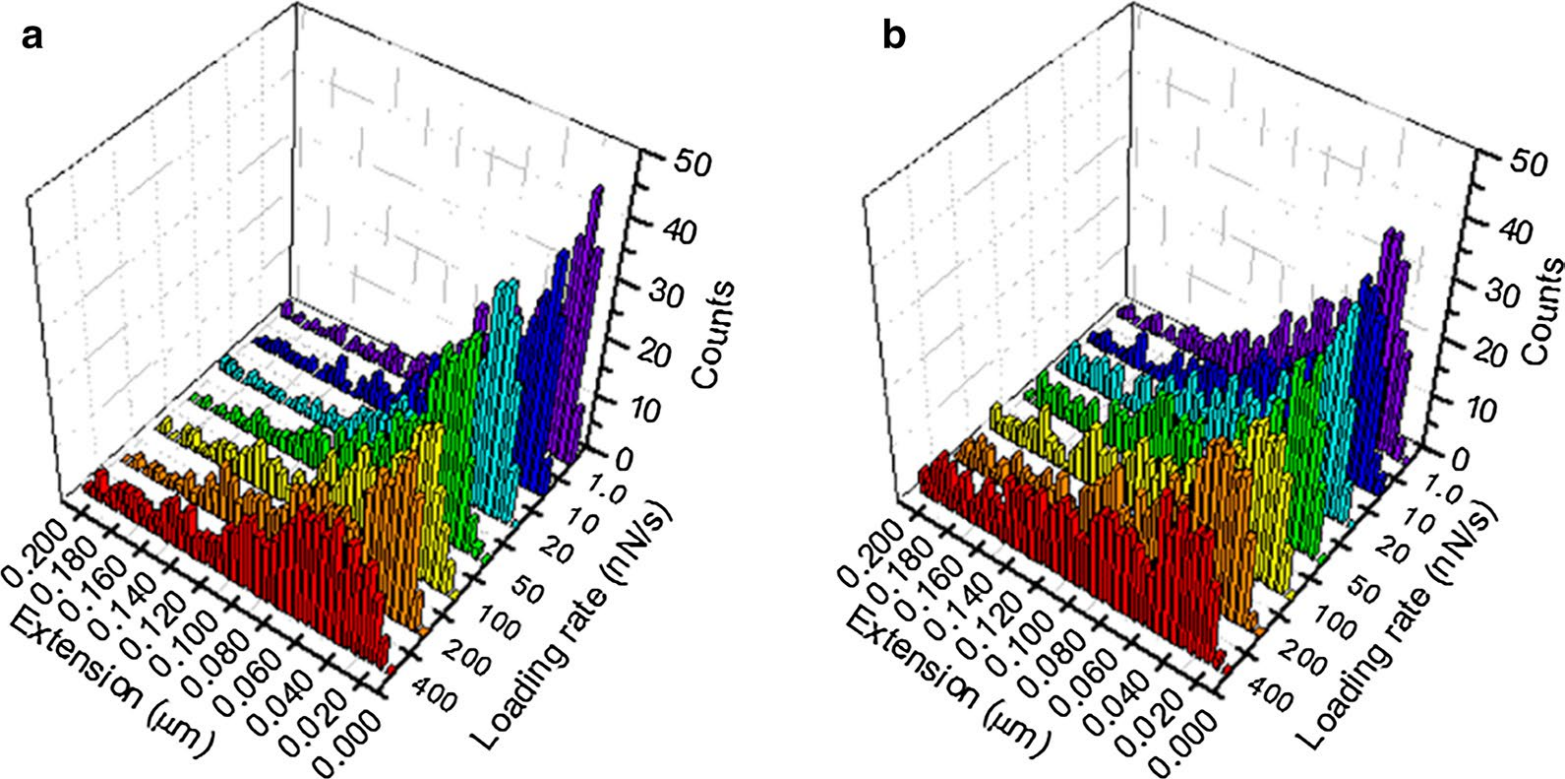
Extension histograms for APT33 (**a**) and APT45 (**b**) in the DFS measurements under seven different loading rates

**Fig. 7:**
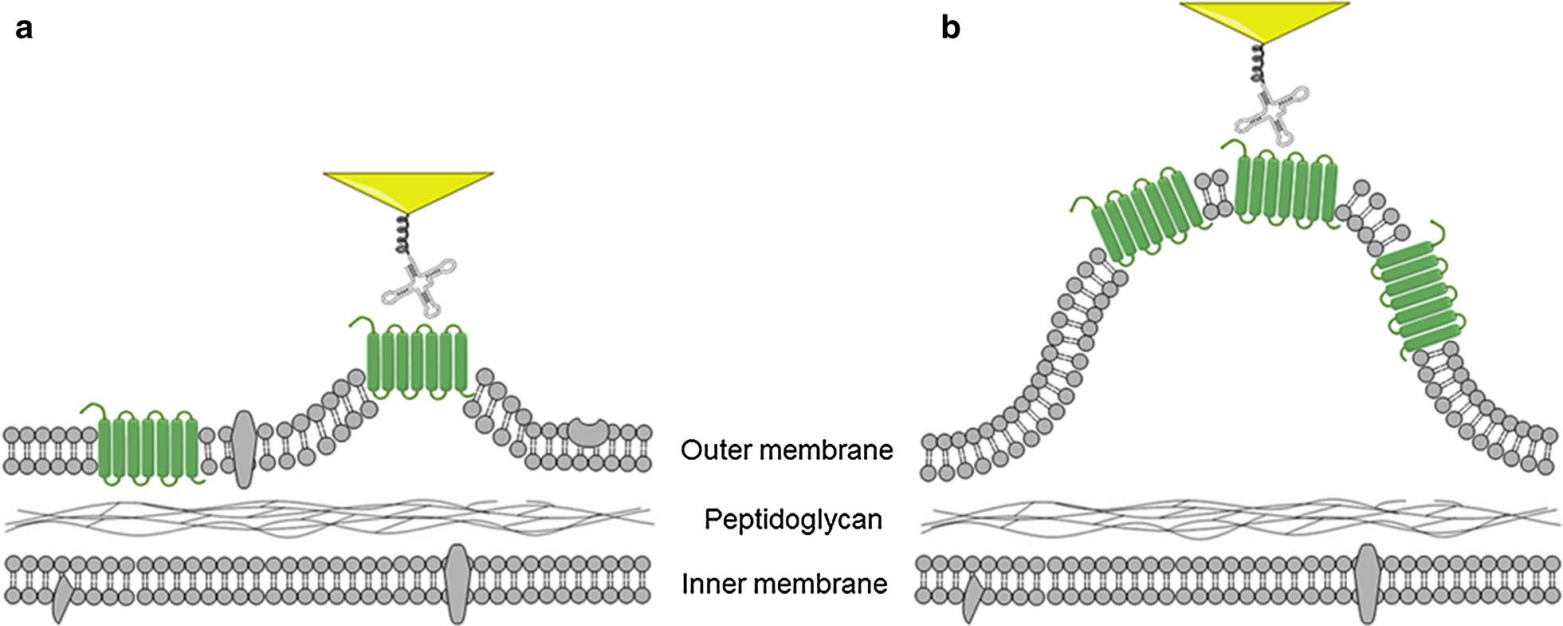
The schematics showing the non-specific interactions between the aptamer on the AFM tip and the OMP on the outer membrane surface during the DFS measurements. **a** During the unbinding process, the lipid molecules (in *gray*) may generate non-specific force to the OMP (in *green*) due to the elastic property of the outer membrane. **b** When the OMP is in the area of the OMV formation, the measured extension can be significant different from other areas

For the extension histograms in Fig. 6, the seven loading rates (R) 1, 10, 20, 50, 100, 200, and 400 nN s^−1^ are shown in the histograms, with the extension distributions of APT33 in (A) and APT45 in (B). Here the extension histograms show clearly different distributions between APT45 and APT33 under the same loading rate. For both aptamers, more peaks appeared under higher loading rates. The major reason is that high loading rates will cause more non-specific interactions between the aptamers and the complex structure of the bacteria outer membrane. For example, the local structure of the OMV formation will introduce complex non-specific extension to the aptamers [23, 27]. The bacteria outer membrane may also generate certain non-specific interactions to the aptamer when external force was applied to its surface. These non-specific unbinding processes have been discussed in literature and shown in Fig. 7 [28, 29]. Another possible reason is that the aptamer may have higher probability to interact with more than one OMP protein under high loading rates, when the elasticity of the local membrane surface lead to the moving and overlapping of multiple OMP molecules. Unfortunately, the detailed structures of the OMPs on the membrane surface are unknown, so the AFM force-extension curves are the only method to provide the information for the reaction mechanism. More structural information is required if we want to have better understanding of the complex relationships among the extension histograms and loading rates.

**Fig. 8:**
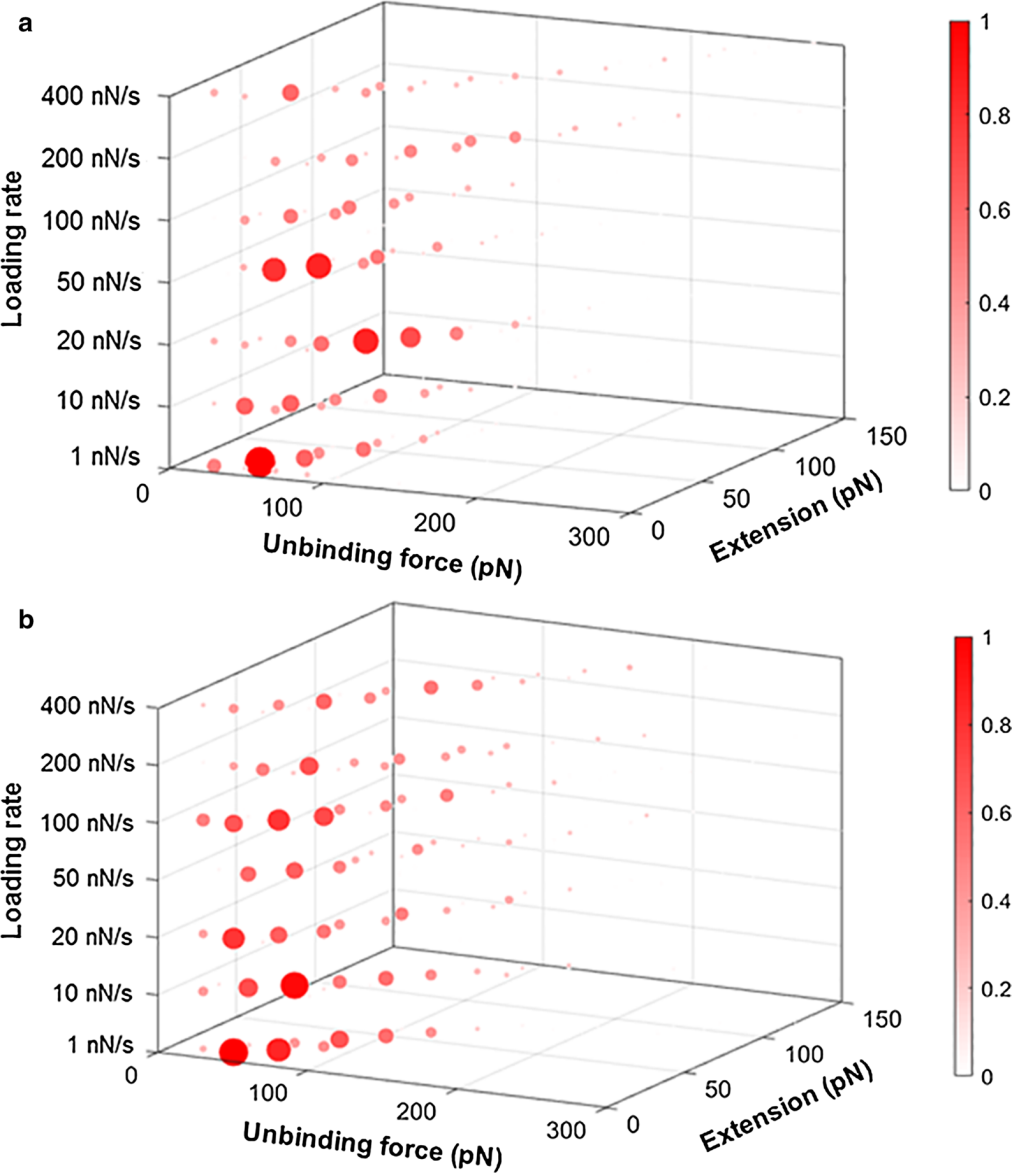
The 4D histograms constructed from force values and their extension values of APT33 (**a**) and APT45 (**b**) under seven loading rates, 1, 10, 20, 50, 100, 200, and 400 nN s^−1^. The *color bars* represent the normalized frequencies for the data points. The different sizes of the data points are proportional to their color

Another method to analyze the force-extension curves is using the 4D histograms to show the correlation between the most probable force values and the most probable extension values. Around 300–400 force values and their corresponding extension values were counted under each loading rate for APT33 (Fig. 8a) and APT45 (Fig. 8b). The data points with higher frequency (normalized) are shown in darker red color according to the color bar, and the size of each data point is also corresponding with its frequency at its certain force, extension, and loading rate coordinate in these 4D histograms. These force and extension values are considered to represent the specific interactions between the aptamer and the bacteria OMPs on the cell surface. The data points colored in lighter red and in smaller size are the coordinates with much less counting frequency, and considered to be caused by non-specific interactions. Some force values may have similar counting frequency and overlay together in conventional histograms in Fig. 4a, b, but can be distinguished by their different extension values in 4D histograms in Fig. 8a, b. Therefore, the 4D histograms can be used to exclude non-specific interactions and reveal the true relationships between force and extension values.

In Fig. 8, most force and extension values follow the similar trend, the proportional increases of both force and extension under the same loading rate (the seven x–y planes along the z-axis), so the data points with high counting frequencies form a dot line expanding along the diagonal within each loading rate. The dot lines also expand with the increase of the loading rates. Several peaks (large data points in dark red) exist within the dot line, and clearly show the most probable force-extension-loading rate correlation area. These most probable force values and extension values were combined together as the major peaks in Fig. 8 to determine the specific interactions. These highlighted correlation areas represent the specific interactions between the aptamer and the OMPs on bacteria surface, since they have high counting frequency. The force-extension-loading rate coordinates of these specific interactions are listed in Table 1. These coordinate values were not obtained by Gaussian fitting, so the most probable force and extension values are different from the ones in Figs. 4 and 6. Nevertheless, some coordinates do reveal consistent trends. For APT33, the force values of 40, 60, and 80 pN have high counting frequencies in the 4D histogram, and their corresponding extension values increased with the increasing of loading rate. For APT45, force values 20, 40, 60, 80, and 120 pN show high counting frequencies with increasing extension values. All of these information are revealed by the 4D histograms, and can be used to distinguish these two aptamers, and exclude the non-specific signals from the large amount of measured data.

**Table 1:** The most probable force and extension values obtained from the 4D histograms of APT33 and APT45 in Fig. 8, under seven loading rates

Loading rate (nN s^−1^)	Peak coordinate values [force (pN), extension (nm)]	
	APT33	APT45
1.0	[40,20], [60,30], [80,50]	[20,20], [40,30], [80,40]
10	[40,30], [60,40], [80,50]	[40,20], [60,30], [80,40], [100,50]
20	[40,40], [60,50], [80,60]	[20,20], [40,30], [60,40], [120,50]
50	[60,20], [80,30], [120,40]	[40,20], [60,30], [80,40], [140,50]
100	[40,30], [80,40], [110,50]	[40,30], [60,40], [100,50], [140,60]
200	[40,50], [80,60], [120,70]	[40,40], [80,60], [120,70]
400	[40,30], [80,50]	[40,30], [60,40], [80,50], [120,60]

**Fig. 9:**
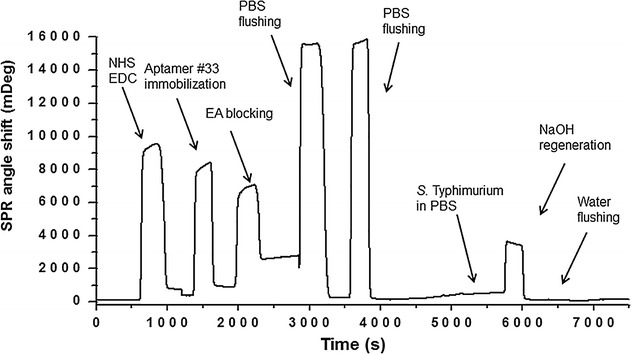
The representative SPR sensorgram for the *S. typhimurium* detection

### Bulk solution measurements using SPR

**Fig. 10:**
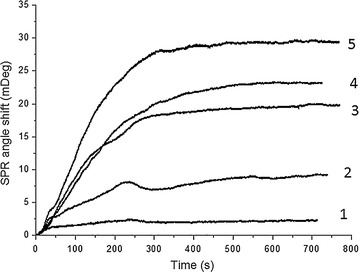
The SPR data obtained under five *S. typhimurium* concentrations. The aptamer APT33 was used as the probe molecule on CD-modified Au(111) surface. The concentrations for *S. typhimurium* are 1: 7.1 × 104 CFU mL^−1^, 2: 7.6 × 106 CFU mL^−1^, 3: 7.6 × 107 CFU mL^−1^, 4: 2.4 × 108 CFU mL^−1^, and 5: 2.4 × 109 CFU mL^−1^

The SPR measurements have been conducted according to the similar protocol previously published (Figs. 9 and 10) [5]. The SPR angle shift signals in the sensorgram include the experimental steps of surface modification with the APT33, the blocking of unoccupied carboxyl groups on CD surface with EA, the binding between aptamer and *S. typhimurium*, and the surface regeneration with NaOH. The SPR angle shift here was generated by the *S. typhimurium* PBS solution with the concentration of 7.6 × 10^7^ CFU mL^−1^. The SPR responses for five *S. typhimurium* concentrations including 7.1 × 10^4^, 7.6 × 10^6^, 7.6 × 10^7^, 2.4 × 10^8^, and 2.4 × 10^9^ CFU mL^−1^ are shown in Fig. 10. These different concentrations were used to estimate the limit of detection (LOD). This experiment indicates that SPR is a promising technique for the detection of *S. typhimurium* in solution, such as food matrix.

**Fig. 11:**
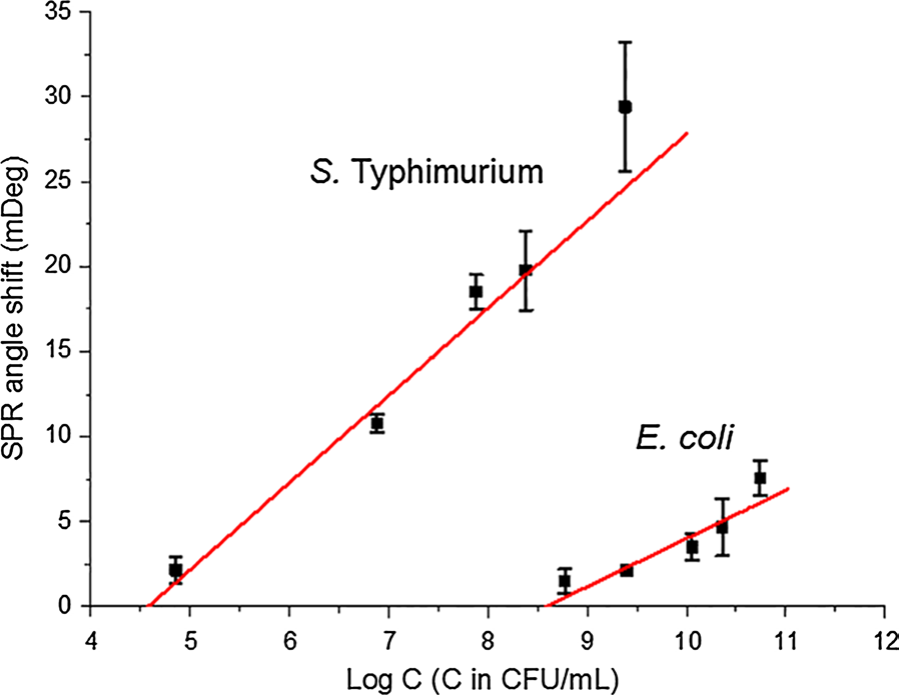
The linear fitting of SPR signals for *S. typhimurium* and *E. coli* samples. The concentrations for *S. typhimurium* are 7.1 × 104, 7.6 × 106, 7.6 × 107, 2.4 × 108, and 2.4 × 109 CFU mL^−1^. The concentrations for *E. coli* are 5.8 × 108, 2.4 × 109, 1.1 × 1010, 2.2 × 1010, and 5.3 × 1010 CFU mL^−1^

The SPR signals from these five concentrations showed good linear correlation in Fig. 11, indicating that SPR can be used for quantitative analysis of *S. typhimurium* in water solutions. In this case, the limit of detection (LOD) is estimated as 3 × 10^4^ CFU mL^−1^. The control sample *E. coli* showed a much higher LOD, around 3 × 10^8^ CFU mL^−1^. Therefore, the aptamer proved to be specific for *S. typhimurium*. This SPR detection is the first step to further develop high-throughput SPRi biosensors for identifying different bacteria and their serotypes in the future.

## Conclusions

The AFM topography and recognition images were obtained in order to prove the specificity of the aptamers to *S. typhimurium*. The quantitative analysis of the interactions between aptamer and *S. typhimurium* under different DFS loading rates was used to estimate the off-rate values for the single-molecule interactions between the aptamers and *S. typhimurium*. The results of this fundamental study are essential for the better understanding of the aptamer and bacteria activities on biosensor surface. The detailed investigation on the force and extension properties of the aptamers binding to the *S. typhimurium* OMPs will also foster the biophysical studies of other bacteria and cells.

The SPR bulk solution measurements have shown the capability of this technique in the detection of *S. typhimurium* samples. The LOD was determined as 3 × 10^4^ CFU mL^−1^ for the *S. typhimurium* in water. The protocols for the surface modification and SPR measurements are the guides for future high-throughput detections of different bacteria serotypes using their corresponding aptamers. Thus, the label-free, high-throughput biosensors have the potential to solve the safety issues in the food industry.

## Abbreviations

AFM: atomic force microscopy
DFS: dynamic force microscopy
APT33: aptamer #33
APT45: aptamer #45
SPR: surface plasmon resonance
OMP: outer membrane protein
OMV: outer membrane vesicles
LOD: limit of detection
3D: three-dimensional
CD: carboxymethylated-dextran
HS-PEG-COOH: thiol-(polyethylene glycol)-acid (M.W. 2000)
EDC: 1-(3-dimethylaminopropyl)-3-ethylcarbodimide hydrochloride
NHS: N-hydroxysuccinimide
